# Using Flies to Understand Social Networks

**DOI:** 10.3389/fncir.2021.755093

**Published:** 2021-12-03

**Authors:** Jacob A. Jezovit, Nawar Alwash, Joel D. Levine

**Affiliations:** ^1^Department of Cell and Systems Biology, University of Toronto Mississauga, Mississauga, ON, Canada; ^2^Department of Cell and Systems Biology, University of Toronto, Toronto, ON, Canada; ^3^International Research Centre for Neurointelligence, University of Tokyo, Tokyo, Japan

**Keywords:** *Drosophila*, neurogenetics, social networks, pheromones, machine vision

## Abstract

Many animals live in groups and interact with each other, creating an organized collective structure. Social network analysis (SNA) is a statistical tool that aids in revealing and understanding the organized patterns of shared social connections between individuals in groups. Surprisingly, the application of SNA revealed that *Drosophila melanogaster*, previously considered a solitary organism, displays group dynamics and that the structure of group life is inherited. Although the number of studies investigating *Drosophila* social networks is currently limited, they address a wide array of questions that have only begun to capture the details of group level behavior in this insect. Here, we aim to review these studies, comparing their respective scopes and the methods used, to draw parallels between them and the broader body of knowledge available. For example, we highlight how despite methodological differences, there are similarities across studies investigating the effects of social isolation on social network dynamics. Finally, this review aims to generate hypotheses and predictions that inspire future research in the emerging field of *Drosophila* social networks.

## Introduction

Collective behavior can be defined as a manifestation of group-level patterns produced by simple interactions between individuals (Sumpter, 2010). Animals display a wealth of interesting collective behaviors such as migrating geese flying in V-shaped formation, flocks of starlings turning in unison, schools of fish splitting and reforming while outmaneuvering a predator, honeybees foraging, and the division of labor in ant colonies (Sumpter, 2010). How individuals organize these interactions depends on their social environment. Several factors, such as the composition and size of the group, alter the social environment and may affect expression of collective behaviors. The African migratory locust illustrates this phenomenon: crowded group conditions alter the morphology, physiology, and behavior of individual locusts, resulting in aggressive swarms (Gillett, 1973). Similarly, manipulating group composition in the fruit fly affects the mating behavior and cuticular hydrocarbon profile of individuals through differences in gene expression (Kent et al., 2008; Krupp et al., 2008; Billeter et al., 2012). These examples, easily seen by the naked eye, emphasize that interactions between individuals defines the social environment, and, in turn, the social environment influences the behavior of the collective group.

The relationship between individual interactions and collective behavior of animal groups can be studied in numerous ways. Simple informative assays have been developed that compute the distance to an animal’s nearest neighbor through static images or video sequences (Simon et al., 2012). More elaborate approaches involve tracking the identity and motion of animals in video recordings with machine vision software (Branson et al., 2009; Grover et al., 2009; Eyjolfsdottir et al., 2014; Crall et al., 2015; Wario et al., 2015; Robie et al., 2017), and this has inspired the application of machine learning algorithms to classify and predict various social behaviors (Kabra et al., 2013). Research on collective behavior of animals often converges on the theme that simple rules applied to pair-wise interactions drive emergent group structures (Mersch et al., 2013; Baracchi and Cini, 2014; Pasquaretta et al., 2016a). Although more remains to be uncovered about how animals form collective units, our understanding has progressed from experiments quantifying social interactions on an individual basis to social network analyses that emphasize the group as an entity.

**FIGURE 1:**
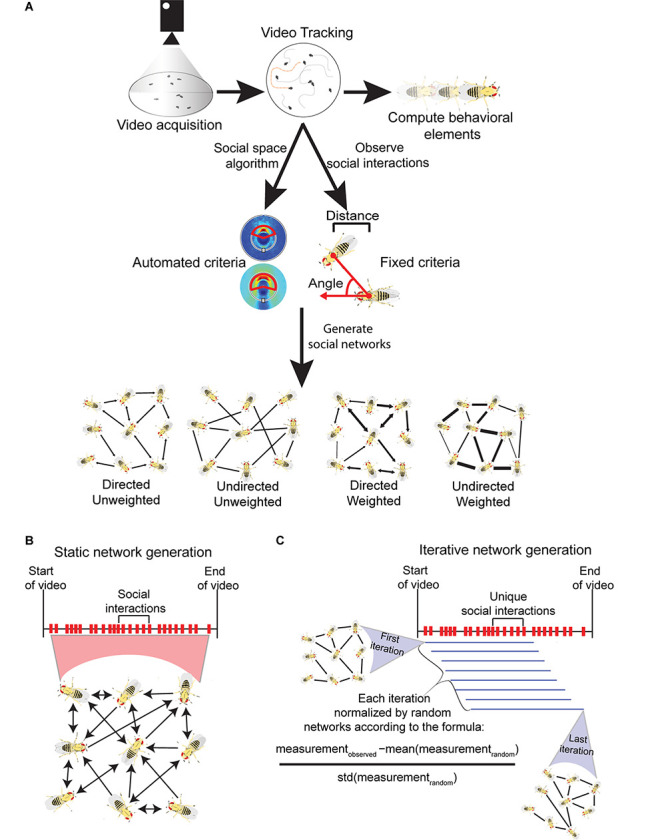
Visualization of the methods involved in acquiring *Drosophila* social networks. **(A)** First videos with a specific number of flies confined in an arena are acquired and the position, orientation and identity of each fly is tracked with machine vision software (e.g., Ctrax). This information acquired from tracking can be used to calculate a variety of behavioral element measures such as the average locomotor activity of the flies. To generate networks, criteria that define a directed interaction are necessary. Typically, three parameters are used: (i) the angle connecting the center of the interactee fly relative to the interactor fly (shown with red arrows); (ii) the distance between the two flies’ center of mass; and (iii) how long these conditions must be maintained for. The criteria can be defined manually, based on observation (fixed criteria) or automatically computed through a published algorithm (automated criteria; see Schneider and Levine, 2014). Once the criteria are selected, social networks can be generated each time they are met in the tracked videos. Networks can be computed with the following properties: (i) directed - the directionality of incoming or outgoing interactions are recorded; (ii) undirected – the directionality of interactions are not recorded; (iii) weighted – interactions are weighted to reflect the strength or frequency of interactions between nodes; (iv) unweighted - networks are binary and only consider the presence or absence of interactions between individuals. **(B)** Visualization of static networks, a conventional form of SNA where every observed social interaction within a video sequence is combined into a single, large social network that encompasses the entire history of social interactions. To avoid saturation of node connections, static networks can be weighted. **(C)** Visualization of the iterative network method (published by Schneider et al., 2012) where a variety of network iterations are generated throughout a single video sequence. Once a threshold number of unique interactions are observed, one iteration is generated. Each subsequent unique interaction creates a new iteration where the oldest interaction is removed. Each iteration is normalized to randomly generated networks with equal degree distributions. All iterations also have the same number of interactions. As a result, degree distribution and density are controlled through this method.

Social network analysis (SNA) relies on statistical tools to identify patterns of interaction in groups and consequences of social structure (Krause et al., 2009). Applications of SNAs originated in the 1930s to study sociological factors of human populations (Moreno, 1934; Lewin, 1951; Wasserman and Faust, 1994; Scott, 2000). Later, SNAs were applied to studying exclusively the social structure of non-human primates (Sade, 1965; Fedigan, 1972; Pearl and Schulman, 1983; Sade et al., 1988; Kudo and Dunbar, 2001; reviewed in Brent et al., 2011). In the last 20 years, SNAs have been applied to various animals in the field and laboratory such as fish (Croft et al., 2004), birds (Boogert et al., 2014), insects (Otterstatter and Thomson, 2007; Formica et al., 2017; Stroeymeyt et al., 2018), and other mammals including spotted hyenas (Ilany and Akçay, 2016), elephants (Goldenberg et al., 2016), and giraffes (Shorrocks and Croft, 2009). Across this literature of animal behavior, a *social network* is defined as any number of nodes interconnected via social ties between them (Krause et al., 2009). *Nodes* are defined as social entities that represent an individual animal. *Edges* represent the connection between two nodes (social relationship or interaction), and these can be *weighted* or *unweighted* (see Figure 1). Unweighted networks are binary and consider only the presence or absence of an interaction between individuals. Weighted networks assign numerical values to all edges in the network, and these values typically reflect the strength or frequency of interactions between nodes. Weighted networks summarize the history and structure of the group and unweighted networks emphasize the distribution of interactions within the group, and each approach has different strengths and limitations. In a *directed network*, edges represent both the connection of nodes and the directionality of an incoming or outgoing interaction. In an *undirected network*, edges represent the sum of all interactions between a pair of nodes but does not take the direction of interactions into account (see Figure 1). Finally, a social network represents connections between nodes over time. Social networks may be *static*, meaning all connections between nodes over a period of time are represented in a single network that represents a history of social connections. Alternatively, *iterative* approaches to networks have been studied. *Iterative* refers to a process of generating multiple transient social networks over a set interval of time to measure dynamic social properties of animal groups (Schneider et al., 2012; reviewed by Blonder et al., 2012; Farine, 2017). Iterative networks offer opportunities to analyze how social connections and group-wide network properties change throughout time.

Both static and iterative social networks derive from pair-wise interactions, which are analyzed to assess pattern and structure. In some cases, network measures describe individual nodes, and in other cases qualities of the entire network. *Degree* is the number of edges connected to a single node. In a directed network, *in-degree* represents the sum of incoming interactions, and *out-degree* represents the sum of outgoing interactions from a single node. Every node in a network has these degree scores, and the *degree distribution* is used to characterize features of a network, such as whether it is random. In a weighted network, the *strength* of a node is calculated as the sum of the edges’ weights connected to that node. Edges are often weighted by the number of interactions between nodes to emphasize short interactions or by the duration of interactions between nodes to emphasize longer social interactions (Bentzur et al., 2020). In a directed and weighted network, the *in-strength* is the sum of the incoming edge weights, and *out-strength* is the sum of the outgoing edge weights. The *density* of the network is defined as the number of actual connections between nodes divided by the maximum number of connections possible between nodes in the network. This measurement indicates how densely individuals are connected throughout the network. There are a variety of properties that measure different aspects of the network. Examples of these properties are listed and defined in Table 1.

**TABLE 1:** A list of common social network measurements defined by their both technical definition and their general applications.

**Network measure**	**Definition**	**Application**	**References**
Degree	Number of edges connected to a single node. In-degree refers to the number of interactions a node receives, and out-degree refers to the number of interactions a node outputs.	In all types of networks, degree informs how popular a single node is toward receiving and/or relaying connections.	Wasserman and Faust, 1994
Strength	In networks with weighted edges, strength is the sum of all edge weights connected to a node. In-strength refers to the sum of all edge weights a node receives, and out-strength refers to the sum of all edge weights a node outputs.	In weighted networks, strength informs overall how popular a single node is toward receiving and/or relaying connections relative to the weight of each connection.	Bentzur et al., 2020
Density	Proportion of actual connections in a network over the number of theoretically possible connections.	Measures to what extent the network connections are filled out between nodes.	Bentzur et al., 2020
Betweenness centrality	Number of shortest paths that traverse a node.	Measures how central a node is in a network for relaying information and maintaining the network cohesion.	Newman, 2010
Weighted closeness centrality	Calculated as inverse between the shortest path between two nodes, from one node to all other nodes in the network and weighted for number of connections among nodes.	Measures how central a node is in a network for relaying information and maintaining the network cohesion.	Pasquaretta et al., 2016a
Eigenvector centrality	Directly related to the number of contacts a node has and to the relative weight of the nodes to which it is connected.	Measures how central a node is in a network for relaying information and maintaining the network cohesion.	Pasquaretta et al., 2016a
Information centrality index	Calculated by combining all the paths present in a network and assigning a weight to them that is equal to the inverse of the path length.	It reflects the amount of information per individual contained in all possible paths that originate from and end with that individual.	Pasquaretta et al., 2016a
Clustering coefficient	A measure of how interconnected nodes are to one another.	Typically used to measure how cliquish nodes are in a network.	Newman, 2010
Modularity	A measure of how a network can be subdivided into clusters of sub-networks.	Typically used to measure how cliquish nodes are in a network.	Pons and Latapy, 2005
Assortativity	A measure of the homogeneity of the degree distribution of a network.	Distinguishes whether nodes in a network all have a similar degree.	Newman, 2010
Global efficiency	A measure of redundant pathways in the overall network and how efficient information can spread.	Distinguishes whether the overall network has shorter or longer paths between nodes.	Latora and Marchiori, 2001

Social network analysis provide researchers with a powerful tool that contributes to our understanding of mechanisms underlying collective behaviors. The aim of SNA across animals has been dedicated to understanding how ecology and evolution affect collective behavior. For instance, there is evidence that wild animals occupy consistent positions in social networks when introduced to new environments (Krause et al., 2017; Canteloup et al., 2020), and across changing seasons (Blaszczyk, 2018; Stanley et al., 2018b; Rose and Croft, 2020). Also, social network structures of animals analyzed in captivity are consistent with those studied in the wild (Brandl et al., 2019; Ripperger et al., 2019), suggesting that there is order to animal social groups that can be predictably recreated and measured using statistical approaches. Other factors of biological relevance are known to influence the social network position of animals such as age (Baracchi and Cini, 2014; Liao et al., 2018), development (Boogert et al., 2014; Brandl et al., 2019) and reproductive success (Oh and Badyaev, 2010; Formica et al., 2012). Social networks also map how a single animal is connected to the larger population and this can offer insight into probabilities of disease contagion (Sah et al., 2018). The ability to relate biological factors to social networks makes SNA an appealing means to further study animal behavior.

Traditionally SNAs were used to study animals in the field, but increasingly more work has emerged that apply SNA to animals in the laboratory. This shift is a result of recent advancements in the automated identification and tracking of individuals (Branson et al., 2009; Straw and Dickinson, 2009; Greenwald et al., 2015; Hong et al., 2015; Robie et al., 2017). Increased interest in applying SNAs to the genetic model organism, *Drosophila melanogaster* has surfaced. Although the number of these studies is currently limited, the research questions addressed are surprisingly diverse. Such studies also provide insight into the social diversity and group-level complexity of these ‘simple’ organisms. However, the SNA approach differs in these studies at the experimental, statistical, and conceptual levels. The aim of this review is to compare the scope, objectives, and methods of these studies, and attempt to draw parallels between them and the broader literature of animal social networks. In the process, we highlight the benefit of *Drosophila* insects toward studying complex social phenomena and we attempt to generate hypotheses and predictions that may inspire future experiments.

## *Drosophila* Social Networks

### Social Space

Social network analysis relies on a concrete definition of social behavior to fill connections between nodes. This definition varies across animal species and the scope of the study. For example, social networks generated from animals in the field often considers individuals socially connected if they are found in a common geographical location (Goldenberg et al., 2016; Deng et al., 2017; Brandl et al., 2019). More precise animal interactions may be used to build social networks and examples include grooming or dominance interactions observed in a variety of mammals (Madden et al., 2009; Blaszczyk, 2018; Büttner et al., 2019). Social networks can also be produced from animals in the laboratory, based on precise social interactions observed or tracked in video sequences. Examples include physical contact between the antennae of ants (Blonder and Dornhaus, 2011), and the transfer of regurgitated food (trophallaxis) observed in bees (Gernat et al., 2018). What forms of social communication occur in *Drosophila*? Decades of investigation into the genetic, neurological, and physiological basis of social behavior in *D. melanogaster* offers the consensus that social communication involves various combinations of visual, acoustic, tactile, and chemosensory cues (von Schilcher, 1976; Agrawal et al., 2014; Bontonou and Wicker-Thomas, 2014). As we will discuss below, social networks in *Drosophila* are derived from physical encounters between conspecifics, like SNA in ants and bees (Blonder and Dornhaus, 2011; Gernat et al., 2018). In this section we discuss the *social space* of flies, defined as spatial criteria between the bodies of flies that approximate social interactions. This can be conceptualized as a physical space that once crossed, scores a social interaction. Also, we note that the terminology in the field is not consistent. Social distance is used by some authors (Simon et al., 2012; Brenman-Suttner et al., 2020) and social space by others (Schneider and Levine, 2014; Montagrin et al., 2018). We favor social space and use it here as a matter of preference, not rigor, since these terms may be used interchangeably.

The first observation of organized spatial positioning in *Drosophila* is credited to Sexton and Stalker, who noticed that groups of female *Drosophila paramelanica* touch one another with their forelegs to maintain uniform spacing at high group density (Sexton and Stalker, 1961). This observation was rediscovered by Schneider et al. (2012) over 50 years later in *D. melanogaster*. Repeated video recordings of flies in a homogenous group revealed ‘touching’ behavior, which involves the foreleg of an ‘interactor’ touching the ariste, head, body, wing, or leg of an ‘interactee.’ Before touching, the interactor would typically approach the side of the interactee’s body at acute angles, unlike in courtship when males tap the rear of a female’s abdomen. This behavior can be classified using three social space parameters: (i) *distance* of the shortest line segment connecting the center of mass between the interacting flies; (ii) *angle* of the line segment connecting the centers of mass of both flies and the line segment protruding from the head of the interactor; (iii) the *time* fulfilled during these touch encounters (Figure 1). Schneider et al. (2012) defined a social interaction between multiple flies as distance ≤ 2 body lengths, angle ≤ 90 degrees, and time ≥ 1.5 s. Since this was repeatedly observed in a social context devoid of courtship behavior, these social space criteria arguably represent the most basic unit of social communication in flies. As flies house gustatory taste receptors within bristles on their legs (Vosshall, 2007), it is possible flies use touch, taste or both as a form of social communication, in addition to visual and olfactory sensory modalities (Vosshall and Stocker, 2007; Zhu, 2013). Additional studies have applied similar criteria for scoring social interactions, with some modification that involved relaxing the angle parameter (Bentzur et al., 2020) and restricting the distance parameter (Dawson et al., 2018; Liu et al., 2018).

Social space criteria defined by Schneider et al. (2012) were derived by observation and applied as a standard across different types of flies. This method did not consider differences in social space criteria that could occur between strains and species. This issue was addressed by the development of an algorithm that analyzes spatial positioning between flies and maps their typical social space in an unsupervised fashion (Schneider and Levine, 2014). More specifically, the algorithm analyzes the spatial positions of every fly in all tracked videos. Then background noise is eliminated by analyzing spatial positions of “virtual trials” which consist of fly tracks randomly sampled from separate videos. With that background subtraction, the algorithm identifies distance, angle and time parameters that are over-represented in videos of flies socially interacting compared to the non-social virtual trials. This can be interpreted as the typical spatial boundary between flies from the analyzed videos. Any fly crossing this boundary within the videos is considered socially interacting. For the remainder of this review, we will refer to social space criteria generated from this algorithm as “automated criteria” and all other criteria derived from human observation as “fixed criteria.”

The social space algorithm was first applied to male and female *Canton-S* and *Oregon-R* strains of *Drosophila melanogaster*. The automated criteria that were computed differed from the previously published fixed criteria (distance ≤ 2 body lengths, angle ≤ 90 degrees, and time ≥ 1.5 s; Schneider et al., 2012). The distance parameters ranged between 1.75 and 2 body lengths, the angle parameters ranged between 115 and 160 degrees and the time parameters ranged between 0.4 and 0.6 s (Schneider and Levine, 2014). Using different methods of image analysis over time, Simon et al. (2012) and Jiang et al. (2020) demonstrated that the average nearest neighbor distance between flies studied in a group converges between 1.5 and 2 body lengths. Other researchers studying group dynamics in *Drosophila* have also applied a distance criterion between 1 and 3 body lengths based on their own observation (Pasquaretta et al., 2016a; Bentzur et al., 2020; Wice and Saltz, 2021). A recent comparative study conducted on 20 drosophilid species found that the average social distance of each species ranges between 1 and 3 body lengths. Additionally, the authors observed that the average leg length of each species relative to their body size positively correlated with social distance. This finding suggests that the variation in the social distance of flies can be explained by their morphology, and it further confirms that 2 body lengths is a reliable social space criterion to capture social encounters between individual flies in a group setting.

When using social space criteria to score the social behavior of flies in a group, it is important to consider how to minimize false-positive interactions. For instance, the automated criteria estimated by Schneider and Levine (2014) displayed an increase in the angle parameters and a decrease in the time parameters compared to the fixed criteria. A wider angle and a shorter time parameter would lead to an increase in the number of interactions, and indeed Schneider and Levine (2014) reported an increase of hundreds of social interactions with the automated criteria. Additionally, false-positive social interactions may occur when two flies, interacting over long periods of time, momentarily slip outside of the social space boundary. This may result in a lengthy interaction between two flies getting counted as several short interactions. Stricter social space criteria have been applied by other researchers, perhaps with the intention of minimizing false-positive interactions. One straightforward approach is reducing the distance parameter so that social interactions are only counted when flies are in close proximity. For example, Liu et al. (2018) recorded interactions exclusively when one fly’s head approached and touched another fly’s rear. Another strategy is the implementation of a ‘gap length’ parameter, which is a set time interval required to elapse before additional interactions between the same pair of flies are counted (Liu et al., 2018; Bentzur et al., 2020). Bentzur et al. (2020) reported that implementing a gap length of 4 s substantially reduced the number of consecutive interactions occurring between the same pairs of flies. Another alternative to filtering excessive interactions is counting subsequent social interactions between unique pairs of flies as done by Schneider et al. (2012). That is, interactions between A and B will not be counted two times in a row. When defining social space criteria, there is a trade-off between filtering false-positive and accepting the loss of true positive interactions and balancing this depends on the researcher. Finally, social space criteria should be redefined if different social contexts are being compared. Flies engaging in aggressive or sexual acts may posture their bodies differently than the touch events described previously and adjusting social space criteria to reflect this may become useful toward future pursuits in the automated behavioral classification of *Drosophila* social interactions.

### Social Networks

Within recent years, there has been increased interest applying SNA to study the sociality of *Drosophila* insects from computational, behavioral, neurobiological, and evolutionary perspectives (Schneider et al., 2012; Schneider and Levine, 2014; Pasquaretta et al., 2016a,b; Liu et al., 2018; Bentzur et al., 2020; Jezovit et al., 2020; Rooke et al., 2020; Alwash et al., 2021). All these studies consist of analyzing video footage tracked by machine vision software and applying social space criteria to generate social networks. Despite differences in the methodology of these experiments (see Table 2), remarkably similar experimental questions have been addressed (see Table 3). In this section, we review recent SNAs applied to *Drosophila.* We compile the various hypotheses tested in these studies and comment on the overlap found in the social network data. We also summarize the SNA methods in these studies and discuss the advantages of each for studying *Drosophila* social networks.

**TABLE 2:** A comparison of all published-to-date *Drosophila* social network studies with their network analysis methods summarized.

**Publication**	**Social interaction criteria**	**Summary of network analysis**	**Group size**	**Length of video recordings**	**Tracking software**	**Post-tracking correction**
Schneider et al., 2012	Time: 1.5 s. Distance: 2 body lengths. Angle: 90°	Unweighted, directed, iterative	12 flies	30 min	Ctrax	Yes (Fixerrors)
Pasquaretta et al., 2016a	Time: 0.5 s. Distance: 1 body length.	Weighted, directed, iterative.	12 flies	4 h	Ctrax	Yes (Fixerrors)
Liu et al., 2018	Touch only: head to tail contact for 0.5 s. Gap length between interactions: 0.5 s.	Weighted, directed, static	16 flies	1 h	Flytracker	No
Bentzur et al., 2020	Time: 2 s. Distance: 2 body lengths. Angle: <0°	Weighted, undirected, static	10 flies	15 min	Ctrax	Yes (FixTRAX)
Jezovit et al., 2020	Automated method (Schneider and Levine, 2014)	Unweighted, directed, iterative	12 flies	30 min	Ctrax	Yes (Fixerrors)
Rooke et al., 2020	Automated method (Schneider and Levine, 2014)	Unweighted, directed, iterative	6 flies, 12 flies, 24 flies	30 min	Ctrax	Yes (Fixerrors)
Alwash et al., 2021	Automated method (Schneider and Levine, 2014)	Unweighted, directed, iterative	12 flies	30 min	Ctrax	Yes (Fixerrors)
Wice and Saltz, 2021	Time: 0.6 s. Distance: <2.5 body length. Angle: <160°	Weighted, directed, static	20 flies	20 min	Flytracker	Yes*

*Authors cross-validated tracking by hand-annotating fly identities in a random sample of 700 frames.

**TABLE 3:** A summary of the research objectives and hypotheses tested in all published-to-date *Drosophila* social network studies.

**Research objective**	**Publications**
Quantification of the emerging properties of *Drosophila* social networks and group formation	Schneider et al., 2012; Pasquaretta et al., 2016a; Liu et al., 2018; Bentzur et al., 2020; Jezovit et al., 2020; Rooke et al., 2020; Alwash et al., 2021
The experimental effects of social isolation on social networks and group formation	Schneider et al., 2012*; Liu et al., 2018; Bentzur et al., 2020
The experimental effects of sensory deprivation on social networks and group formation	Schneider et al., 2012; Bentzur et al., 2020; Rooke et al., 2020
Analysis of social space	Schneider and Levine, 2014
Diffusion analysis - modeling spread of information flow between flies	Pasquaretta et al., 2016a,b
The experimental effects of density and group size on social networks	Rooke et al., 2020
Investigating the evolutionary factors of social networks and group formation	Jezovit et al., 2020; Wice and Saltz, 2021
Genetic underpinnings/heritability of social networks and group formation	Alwash et al., 2021; Wice and Saltz, 2021
Investigation of social networks from mixed groups	Pasquaretta et al., 2016a; Bentzur et al., 2020; Wice and Saltz, 2021

**FIGURE 2:**
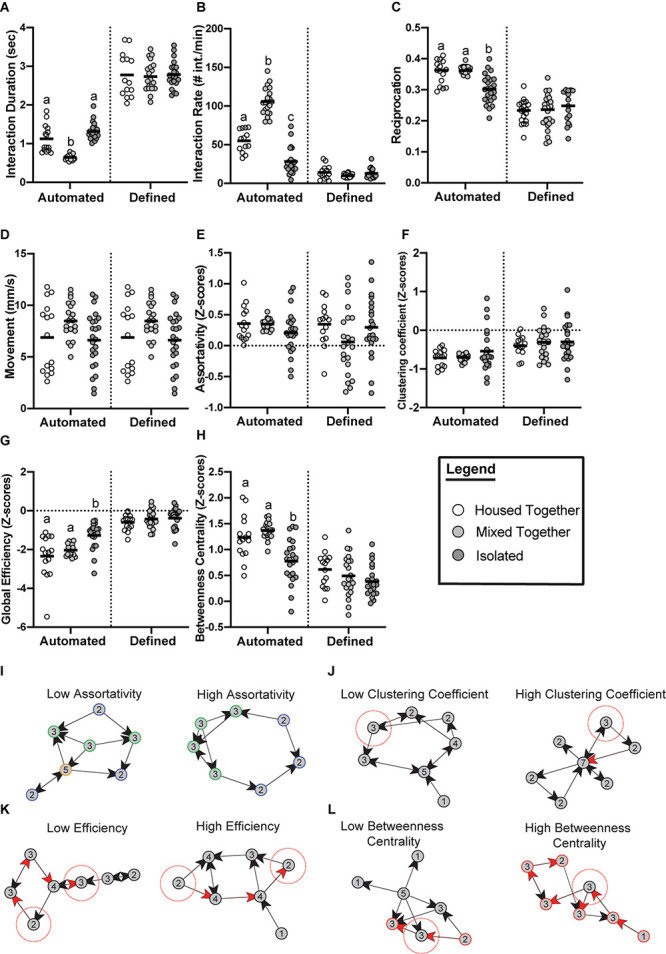
Emerging properties of social networks after social isolation. Data from Schneider et al. (2012) re-analyzed with automated criteria compared to the original published data with fixed criteria reveals social experience significantly affects social interaction measures **(A–D)** and social network measures **(E–H)**. Flies were divided into three treatments: (i) Housed together (white) meaning all 12 flies in one video trial were raised together (*n* = 15 trials); (ii) Mixed together (light gray) meaning all 12 flies in one video trial were unfamiliar with each other from being raised with other flies (*n* = 22 trials); (iii) Isolated (dark gray) meaning all 12 flies in one video trial were completely socially isolated since eclosion (*n* = 24 trials). **(A)** Flies of the mixed group have significantly lower average interaction duration when analyzed using the automated criteria (*p* ≤ 0.0001). **(B)** Flies of the isolated treatment have significantly lower rates of interaction when analyzed using the automated criteria (*p* ≤ 0.0001). **(C)** Average proportion of interactions reciprocated were significantly lower in the isolated groups when analyzed using the automated criteria (*p* ≤ 0.0001). **(D)** Movement did not significantly differ between the three treatments (*p* = 0.0909). **(E)** No significant differences between the three treatments were observed for assortativity when analyzed using the automated criteria (*p* = 0.1027). **(F)** No significant differences between the three treatments were observed for clustering coefficient when analyzed using the automated criteria (*p* = 0.9540). **(G)** Groups of isolated flies form networks with a significantly higher global efficiency compared to controls when using automated criteria (*p* ≤ 0.0001). **(H)** Groups of isolated flies form networks with a significantly lower betweenness centrality compared to controls (*p* ≤ 0.0001). Panels **(A–H)** were analyzed with one-way ANOVA with ranks to determine if statistical differences exist between the groups. Outliers were removed from all the datasets. Bars indicate mean. Letters indicate statistical significance. **(E–H)** Networks were generated from the following automated criteria: distance = 1.5 body lengths, angle = 115°, time = 0.55 s (housed-together); distance = 1.5 body lengths, angle = 110°, time = 0.5 s (mixed-together); distance = 1.5 body lengths, angle = 110°, time = 0.95 s (isolated). Measurements were standardized using *z*-scores as described by Schneider et al. (2012). Panels **(I–L)** defines and visualizes the network measurements analyzed [taken from Schneider et al. (2012)]. **(I)** Assortativity is the correlation between nodes of a similar degree (degree shown as number inside node). Low assortativity indicates nodes of a dissimilar degree tend to interact whereas high assortativity indicates more nodes of a similar degree tend to interact. **(J)** Clustering coefficient reflects the interconnectedness of the nodes in a given network. Networks with low clustering coefficient have a higher proportion of nodes (see focal node highlighted in red) with neighbors that are unlikely to interact. Networks with high clustering have a higher proportion of nodes (see focal node highlighted in red) whose neighbors are interconnected. **(K)** Global efficiency of a network is a measurement of the average shortest path length that information would flow through. Networks with a low efficiency score indicates less efficient information flow on average because the connections between nodes require more steps (visualized by 4 steps required for information to reach the two highlighted nodes through red arrows). Networks with high efficiency have less distances on average between nodes (visualized by 3 steps required for information to reach the two highlighted nodes through red arrows). **(L)** Betweenness centrality is a measure of how many shortest paths traverse a node, which can indicate the relative importance of a node for information flow. Networks with low betweenness centrality have fewer nodes that are critical for network cohesion. This is visualized by the node highlighted with the red dotted circle; this node can easily be bypassed. In the example network with high betweenness centrality, the node highlighted with the red dotted circle cannot be bypassed for information to travel through the network, and networks with high betweenness centrality have more central nodes like that.

*See Figure 2 for re-analyzed data.

#### Video Acquisition and Tracking

First, the precision of social network data depends on reliable, error-free video tracking. The number of errors accumulated by video tracking is dependent on the level of contrast between the flies and the arena background, the length of the videos, and the number of flies (Robie et al., 2017). The most common tracking platform across the *Drosophila* social network literature is Ctrax, an open-source machine vision tracker (Branson et al., 2009). An inconvenient limitation of Ctrax is the requirement to tediously review the tracking data for errors that involve, for example, inconsistent identification of the same individual fly or changes in the size and orientation of the tracks. Each of these errors requires manual review and correction. In a recent experiment that repeatedly filmed 10 flies in an arena for 15 min, an automated error fixing script was applied to edit the tracking errors from Ctrax (Bentzur et al., 2020). An alternative called Flytracker, has been developed that claims to produce error-free tracking (Liu et al., 2018). Recently, Wice and Saltz (2021) cross-evaluated the performance of Flytracker with manual annotation of fly identities from 700 random frames and reported a strong correlation between automated tracking and manual tracking. While these alternatives may increase the speed of data collection, there is always the danger of harboring tracking errors that could lead to a loss of precision and integrity of the SNA. When considering a video tracking pipeline, the speed versus the precision should be weighed appropriately depending on the research objectives. For example, if social networks are generated from interactions defined by distance and angle parameters between flies, then it may be worth thoroughly reviewing and fixing tracking errors that swap identities, and that alter the size and orientation of the fly tracks, as done by multiple studies (Schneider et al., 2012; Jezovit et al., 2020; Rooke et al., 2020; Alwash et al., 2021). If the objective is to generate social networks from interactions defined exclusively by the distance between flies, errors in the orientation of the tracks, for example, can be tolerated and ignored.

#### Static and Iterative Network Generation

Three recent studies analyzed *Drosophila* social networks using the more conventional static network approach (Liu et al., 2018; Bentzur et al., 2020; Wice and Saltz, 2021). This method generates a single network that represents the entire history of social interactions within a single video (visualized in Figure 1). The number of connections within these networks varies depending on the number of interactions observed. All three of these studies weighted the networks, offering additional information on the strength of connections. Four other *Drosophila* social network studies published to date utilized a dynamic iterative approach (Schneider et al., 2012; Jezovit et al., 2020; Rooke et al., 2020; Alwash et al., 2021). This method, published by Schneider et al. (2012), generates directed and unweighted iterations of networks in groups of flies. Unlike static networks, the iterative approach generates multiple networks from a single video at a controlled network density from a sliding boxcar filter (Kossinets and Watts, 2006; visualized in Figure 1). To summarize, one network iteration is built exclusively from a threshold number of unique interactions. When an additional unique interaction is observed, the oldest unique interaction is removed from the network and the newest interaction is added and this forms the second network iteration. This pattern continues and can produce hundreds or thousands of social network iterations in a single video, all offering snapshots of changing network structure over time. To score and compare the network measures of different types of fly groups, each iteration is standardized to thousands of random network permutations with equal in-degree and out-degree distributions. This normalization by degree distribution is then followed by averaging all iterations to summarize the network measure to a single data point. The result is an averaged *z*-score of all network iterations per video. This use of the *z*-score normalization attempts to evaluate properties of the group-wide behavioral interaction patterns independent of the observed individual interaction patterns (degree distribution). Overall this iterative method removes the confounds of network density and degree distribution when comparing networks across different treatment groups (Schneider et al., 2012).

The static and iterative methods each have their advantages and disadvantages (see Table 4). An advantage of the static network approach is that the results are intuitive, whereas the iterative approach is far more abstract to interpret. For example, in a static network, the betweenness centrality score (defined in Table 1) can be compared to each network node and the node with the highest score can be interpreted as being critical to the cohesion of the network. In the iterative approach, every node’s score is averaged in each network iteration and all iterations are averaged. As a result, the iterative analysis sacrifices information about the individual fly in exchange for measuring the overall group. Another distinction between the two methods lies in the network density. The iterative method controls the density of networks by capping the number of social interactions per network and analyzing iterations of density-controlled networks. It was found that iterative networks capped at a 25% network density for groups of 12 flies, which are networks consisting of 33 unique interactions, were more robust than other network densities (Schneider et al., 2012). The static approach simply allows all social interactions in a single video trial to fill out into a single network and therefore network density may vary between video trials. However, network density can be an informative behavioral measure of the animal group since denser networks indicate more social activity and this is not directly measured through the iterative approach. Instead, to gauge differences in network density in the iterative approach the researcher can compute the number of iterations, which is associated with higher social activity. Finally, the static and iterative approaches may be combined as seen in Pasquaretta et al. (2016a) where static networks were generated every 15 min from multiple hours of footage. This combines the simplicity of the static network methods with the advantage of measuring dynamic group activity over multiple time points.

**TABLE 4:** Summary of the advantages and disadvantages involved in simplistic network analyses with fewer parameters (less information column) compared to more complex analyses that require more input but controls more confounds (more information column).

**Factor**	**Less information**	**More information**
Interaction definition	Fixed: • Assumes all individuals and social treatments interact in the same manner. • Can use published criteria.	Treatment-specific: • Requires criteria for all experimental treatments. • Ability to control for differences in interaction patterns when looking at group-level phenotypes.
Directionality of interaction	Undirected: • Assumes any interaction is bidirectional.	Directed: • Assumes interactions are directional.
Value of interaction	Unweighted: • Assumes many interactions between two individuals are as important as a single interaction. • Straightforward methods for network-permutations.	Weighted: • Keeps track of “strong” vs. “weak” interactions, be it time spent interacting or number of interactions. • Permutation methods often fail with small networks.
Network definition	Static: • The network is the accumulation of all interactions over the experimental period. • If structure changes over time, this can be hidden. • If un-saturated, comparisons between different network densities introduce confounds between density and organization of the network.	Iterative: • Can handle arbitrarily long experimental timeframes. • Requires a ‘density’ cut-off value.
Data normalization	Standardized *Z*-score: • Normalizes all measures to a standard scale. • Allows plotting various network measurements on the same axis. • Does not control for anything beyond measurement units.	Network permutation Z-score: • Usually done by generating randomized networks with a controlled network feature (e.g., degree distribution) and standardizing observed networks to null distribution of random networks. • Takes individual-level interaction propensities into account. • Provides unbiased measures of network organization.

#### Social Experience

While the methods of generating and analyzing *Drosophila* social networks differ, one question many of the studies address is how social experience affects the group dynamics of flies (Schneider et al., 2012; Liu et al., 2018; Bentzur et al., 2020). Schneider et al. (2012) examined the effects of 3-day social isolation by measuring the network properties of groups of flies that were all separately reared in isolation, compared to groups of flies reared in a socialized environment. There were no significant differences found in the average network measures between these treatments (Schneider et al., 2012). A limitation to this study was the use of the same fixed criteria (2 body lengths, 90 degrees, and 1.5 s) for the isolated flies and socialized flies. This did not consider potential differences in the social interaction patterns of isolated versus socialized flies. Therefore, to better understand how social experience influences the behavior of flies, we re-analyzed the Schneider et al. (2012) data using automated criteria (Figure 2). Indeed, we find that socially isolated flies tend to engage in longer social interactions than socialized flies (housed-together treatment). On the other hand, a treatment of socialized flies that were combined from separate housing groups (mixed-together treatment) tend to have a shorter interaction time compared to the housed-together treatment. We then generated social networks using the iterative approach and found that social isolation significantly affects the network structure. For example, global efficiency (defined in Table 1) is significantly higher in isolated flies, indicating that isolated flies have more redundant connections in their networks. Isolated flies also display a significantly lower betweenness centrality, indicating that there are fewer central individuals serving as a hub in the network. Across all measures, we observe greater variability in networks of isolated flies compared to the controls, particularly in assortativity and clustering coefficient (defined in Table 1). Lack of social experience in these groups of isolated flies may be contributing to these less predictable network measures. Other behavioral measures, such as the average interaction rate and percentage of interactions reciprocated, were also significantly lower in groups of isolated flies. The new analysis shown here underscores the importance of the automated criteria and makes the findings of Schneider et al. (2012) consistent with recent studies that have addressed these questions in other ways (Liu et al., 2018; Bentzur et al., 2020).

Liu et al. (2018) took a different approach to the same question. Rather than isolate virgin flies for 3 days, 9-day old flies were isolated for 6 days. Replicates of static, directed, and weighted social networks were generated from multiple video sources and then averaged. Liu et al. (2018) revealed groups of 16 flies that had been isolated tend to be more active, interact more often and produce networks with a higher clustering coefficient than groups of socialized flies. Additionally, a time course of 1-day to 6-day long isolation treatments showed that the average clustering coefficient is significantly greater than that of socialized flies at all time points. This suggests that a single day of isolation is sufficient to alter the clustering coefficient of flies, and this may be robust since we also report a higher clustering coefficient in isolated flies (Figure 2). In fact, Liu et al. (2018) also report a higher global efficiency in isolated flies, which also agrees with our re-analyzed data (Figure 2). This similarity illustrates that network measures are robust in flies of different ages since groups of 3-day old flies raised in isolation produce similar networks to 9-day old flies.

A more recent experiment by Bentzur et al. (2020) also found social isolation affects fly networks. Like Schneider et al. (2012), these authors collected flies as virgins and isolated or socialized them for 3 days before recording their behavior. In this study, the authors generated static networks, and they were analyzed two ways: (1) weighting nodes by the number of interactions to emphasize short and acute social patterns; (2) weighting nodes by the length of interactions to emphasize long-lasting social interaction patterns. The authors found that isolated flies displayed a lower average betweenness centrality than socialized flies in networks weighted by the number of interactions and the length of interactions, and our re-analyzed data further validates this finding (Figure 2). Bentzur et al. (2020) also found that, on average, isolated flies have a lower modularity score (defined in Table 1) in networks weighted both ways, indicating that isolated flies produce networks that are less compartmentalized. This can be attributed to their finding that social isolation leads to flies being more active as measured by increased velocity, and decreased instances of flies physically clustering and interacting over long periods. However, higher locomotor activity may lead to more frequent social interactions, resulting in a higher network density, and a higher average degree/strength. Indeed, this was reported in isolated flies (Liu et al., 2018; Bentzur et al., 2020) and may serve as a confound when assessing network structure. Alternatively, the aggregate clustering observed in socially experienced flies could reduce the number of social interactions, skewing the data to a lower network density. Interestingly, in networks weighted by the length of interactions, Bentzur et al. (2020) found no significant difference in the network density between isolated and experienced flies. Perhaps measuring networks weighted by length of interactions reduces the confounds that arise from differences in locomotor activity and frequency of social encounters because these networks favor connections between flies that spend longer periods of time socializing.

Despite the differences in methodology, three studies overlap in showing how social experience affects the group dynamics of flies. Two recent publications were the first to report these effects (Liu et al., 2018; Bentzur et al., 2020), and re-analyzing data from Schneider et al. (2012) further validates these two independent studies. Flies isolated for 3 days form social networks with a lower betweenness centrality (Bentzur et al., 2020) (Figure 2H). This results in less cohesive social networks with fewer central individuals holding the group together. Additionally, isolated flies form networks with a lower modularity (Bentzur et al., 2020), which indicates social isolation leads to less complex network structures. Taken together, these studies show that isolating flies hinders their ability to socialize within groups. This appears to contradict the finding that flies isolated for 1–6 days form networks with a higher clustering coefficient (Liu et al., 2018) (Figure 2F), indicating isolated flies on average may form cliquish groups. However, an automated classifier trained to detect instances of multiple flies physically aggregating found that isolated flies aggregate less than socialized flies (Bentzur et al., 2020). This illustrates the point that social networks capture patterns not necessarily intuitive to the human eye and future experiments would benefit by applying machine learning classifiers to measure additional qualities of social interactions. Measuring a wealth of behavioral classifiers, as done by Bentzur et al. (2020), would help validate and interpret the more abstract social network measures. Another recent experiment by Sun et al. (2020) studied the social attraction of free-walking flies by measuring their proximity to immobilized flies in arenas. With this assay, the authors found evidence that isolated flies exhibited a decrease in social attraction when compared to socialized flies. Finally, we find evidence that isolated flies are just as active as socialized flies and engage in fewer social interactions on average (Figure 2), which contradicts other studies (Liu et al., 2018; Bentzur et al., 2020). This highlights the benefit of automated criteria for generating social networks (Table 4). The behavior of the flies filmed may fluctuate based on experiments being completed at different times. The automated algorithm (Schneider and Levine, 2014) can take these behavioral fluctuations into account and estimate social space criteria reflective of the flies’ behavior in the current experiment. Additionally, automated criteria can take behavioral differences between experimental treatments into account. For example, if socially isolated flies tend to interact differently than socially experienced flies, the automated criteria can correct for this and generate social networks that best represent the social environment being measured.

#### Effect of Density and Group Size on Social Networks

Each study that compared social networks of isolated and socialized flies examined groups of different sizes. A recent experiment by Rooke et al. (2020) demonstrated that group size affects features of social networks by comparing groups of 6, 12, and 24 flies across three different arena sizes. First, the authors found that the average locomotor activity of flies was similar across different group sizes and arena sizes, suggesting flies regulate their movement to compensate for decreased space. In terms of the social networks, the authors generated iterative, unweighted, and directed networks at controlled network density as published by Schneider et al. (2012). Rooke et al. (2020) found that groups of 6 and 12 flies form networks with a significantly lower average clustering coefficient than groups of 24 flies, and this was consistent across three different arena sizes. Additionally, groups of 12 and 24 flies form networks with a significantly higher average betweenness centrality than groups of 6 flies. This suggests that larger groups, on average, have more flies that are central and maintain greater cohesion across the group. Although the number of social interactions increases as the arena size and group size increase, properties of the social networks remain consistent across the same group size. Since the social networks were all generated at a controlled network density, and all flies were reared with equal social experience, differences in the network measures can be attributed to differences in group size. No matter how confined or dispersed a group of flies may be, the properties of the group shift only when the size of the group changes. Perhaps individual flies may be sensitive to changes in group size based on visual feedback and through the perception of pheromone concentration and organize themselves in the group according to these signals.

#### Sensory Modalities and Group Formation

With *D. melanogaster* being one of the most popular organisms for behavioral genetic experiments, the wide availability of mutant strains and genetic tools to manipulate gene expression have been applied to social network experiments. To date, social networks have been generated for flies with disrupted visual, olfactory, gustatory, and acoustic modalities (see Table 5). Schneider et al. (2012) reported that the gustatory mutant *poxn*^ΔXBs6^ displayed an extreme reduction in the ability to form social networks (Schneider et al., 2012). More specifically, 40% of the videos filmed of these mutants did not harbor enough social interactions to form a single iterative network (Schneider et al., 2012). Recently, Jiang et al. (2020) also reported that *poxn* mutants, in addition to a range of other gustatory mutants, displayed an impaired ability to form physical social clusters. Together, this suggests chemosensory receptors are crucial for maintaining the sociality of flies.

**TABLE 5:** Summary of various genes and sensory manipulations studied in *Drosophila* social network experiments.

**Mutation/gene**	**Role**	**Network findings**	**References**
*orco*	Olfactory mutation.	Reduction in the ability to form networks.	Schneider et al., 2012
*Iav* ^1^	Hearing impaired mutation.	No effect on social network measures.	Schneider et al., 2012
*poxn^Δ^ ^*XBs*6^*	Gustatory mutation.	Reduction in the ability to form networks.	Schneider et al., 2012
*w* ^1118^	Mutation associated with neurological and visual defects and reduced life span.	Increased global efficiency.	Schneider et al., 2012
*lush*	Olfactory binding protein that is sensitive to male pheromones.	*lush-*inhibited fly networks increased clustering coefficient and betweenness centrality values in groups of 12 and 24 flies.	Rooke et al., 2020
*foraging*	Pleiotropic gene that influences several metabolic, physiological, behavioral (foraging) and developmental phenotypes.	The rover allele had higher global efficiency values while sitter allele had higher clustering coefficient and assortativity values.	Alwash et al., 2021
*or65a*	Olfactory receptor neurons that mediate chronic responses to male-specific pheromone cVA.	*Or65a-*inhibited fly networks had increased strength and decreased betweenness centrality values, along with reduced modularity.	Bentzur et al., 2020
*or67d*	Olfactory receptor neurons that mediate acute responses to male-specific pheromone cVA.	Inhibition of *or67d* neurons did not influence social networks.	Bentzur et al., 2020
*cyp6a20*	Associated with increased aggression.	Networks with a mixture of WT and *cyp6a20-*knockdown mutants leads to a reduction in betweenness centrality values.	Bentzur et al., 2020

Schneider et al. (2012) also demonstrated that hearing-impaired *inactive* mutants (*iav*^1^) produced social networks that were not significantly different from wild-type flies. Surgical removal of ariste to ablate auditory perception in flies also had no effect on social clustering behaviors (Jiang et al., 2020). However, Jiang et al. (2020) reported that *iav*^1^ mutants form more dispersed social clusters, unlike wild-type flies that are more tight-knit. This is also reflected in social space criteria for *iav*^1^ mutants where the distance parameter was estimated to be larger than wild-type flies (Schneider and Levine, 2014). Although auditory mutants may socially interact and cluster less than wild-type flies, there is currently no evidence that manipulating auditory cues within a group of single-sex flies affects measures of social network structure (Schneider et al., 2012).

To disrupt vision, experiments have been conducted on flies in the dark. Schneider et al. (2012) reported that groups of flies filmed in the dark display a lower clustering coefficient and higher betweenness centrality, but these effects were not considered significant when accounting for multiple test correction (Schneider et al., 2012). Bentzur et al. (2020) found that groups of socially isolated flies behave more similarly in the light and dark compared to socially experienced flies. The authors reported that in networks of socially experienced flies, visual disruption leads to a significantly lower average betweenness centrality, opposite of what was reported by previous studies (Schneider et al., 2012). Despite disagreement in the social network data when subjecting flies to darkness, multiple studies report similarities in how flies aggregate and physically cluster. Using automated behavioral classification, Bentzur et al. (2020) reported that groups of flies in the dark aggregate less often and for shorter periods of time on average. Data by Jiang et al. (2020) also found that wild-type flies in the dark, along with *norpA33* visual mutants, cluster together less than wild-type flies. These two recent studies reinforce observations by Schneider et al. (2012) that darkness decreases the average interaction duration among groups of flies.

Arguably olfaction is the dominant sensory mechanism *Drosophila* depends on to locate foraging sites and conspecifics. Ablating olfaction is complex because *Drosophila* insects possess multiple olfactory receptors that are encoded by multiple genes. The olfactory mutant, *orco*, is known to have a severe loss of smell because it is deficient for a co-receptor that complexes with a variety of odorant receptors (Vosshall and Hansson, 2011). Social networks of *orco* mutants have been shown to have a significantly lower global efficiency than wild-type flies, with *orco* heterozygotes displaying an intermediate score (Schneider et al., 2012). This may indicate that the copy number of the *orco* gene leads to social interactions that, on average, result in a greater social distance between individuals in the network. In the same study, the *orco* mutants displayed a higher clustering coefficient and a higher assortativity compared to controls, although the differences were not statistically significant after multiple test correction (Schneider et al., 2012). Also, the *orco* mutant aggregates less with conspecifics compared to wild-type flies (Jiang et al., 2020). Overexpressing an *orco* transgene in the olfactory system of these mutants led to the flies aggregating like wild-type flies (Jiang et al., 2020). This is similar to an observation of ant *orco* mutants that displayed a reduction in their ability to follow pheromone trails and cluster with other ants (Trible et al., 2017). This cross-species reduction in aggregation suggests that olfaction is crucial for the sociality of numerous insects, and it is no surprise that olfactory mutants produce social networks different from wild-type flies.

#### Behavioral Genetic Studies on Group Formation

In addition to studying the social behavior of fly mutants, the *Drosophila* model system offers genetic tools to manipulate the expression of genes in a tissue-specific manner through the GAL4-UAS system (Elliott and Brand, 2008). This system was applied to recent social network studies to examine the downstream behavioral effects of ablating specific olfactory sensing cells (Bentzur et al., 2020; Rooke et al., 2020). One experiment examined the social networks of flies where the olfactory receptor neurons Or65a and Or67d were inhibited by driving the expression of *kir2.1* in those cells. These olfactory receptors are known to be sensitive to cVA, a male-specific pheromone that mediates aggressive and copulatory behaviors in male flies (Bontonou and Wicker-Thomas, 2014). Interestingly, flies with inhibited Or67d neurons did not produce social networks drastically different from wild-type flies despite there being evidence that Or67d plays a role in social attraction (Bentzur et al., 2020; Sun et al., 2020). However, the inhibition of Or65a neurons leads to a significantly decreased average betweenness centrality (Bentzur et al., 2020). Another experiment focused on inhibiting the olfactory support cells that express the gene *lush*, which is expressed in trichoid sensillae of flies and aids in the binding of ligands to olfactory receptors (Rooke et al., 2020). By driving the expression of *kir2.1* in all *lush*-expressing cells, Rooke et al. (2020) found that *lush*-inhibited flies produce social networks different from controls in larger group sizes. More specifically, groups of 6 *lush*-inhibited flies formed social networks with an average clustering coefficient and betweenness centrality that resembles the wild-type controls. However, in groups of 12 and 24, the *lush*-inhibited flies formed social networks with a significantly higher betweenness centrality and clustering coefficient than wild-type controls (Rooke et al., 2020). Together, results of these studies indicate that different olfactory genes, expressed in different tissues, may play different roles in regulating group-wide social connections in flies.

Transgenic tools have also been used to manipulate the *foraging* (*for*) gene in a recent SNA study. This gene expresses natural polymorphisms in flies that influence behavioral phenotypes in the larval stage called rovers and sitters (Sokolowski, 1980). Alwash et al. (2021) demonstrated that networks of adult rovers and sitters form different social networks, suggesting this gene influences the behavior of adult flies. Sitter flies were shown to display a higher interaction duration and were more likely to reciprocate interactions, whereas rover flies were more active and displayed higher interaction rates. Compared to rovers, sitters formed networks with a higher assortativity and clustering coefficient, as well as a lower global efficiency suggesting there is less efficient information flow within these groups of flies. Alwash et al. (2021) also used separate transgenic lines, generated by Allen et al. (2017; see for details), that carry 1 copy, 2 copies and 4 copies of the *for* allele, respectively. By comparing social networks across these lines, it was found that *for* gene dosage affects the average assortativity, clustering coefficient and global efficiency measures. Additionally, the average interaction duration, the average rate of interactions, the proportion of interactions reciprocated and the activity of flies all changed across different dosages of *for*. The authors confirm that many of the social network differences observed between rovers and sitters are influenced by the *for* locus. These findings characterize the influence of a specific gene on social network dynamics in *Drosophila*, shedding light on the genetic underpinnings of sociality.

Multiple independent experiments that measured the social behavior of *Drosophila* mutants and transgenic flies with inhibited neurons revealed that sociality of flies is multisensory. In unisex groups of *D. melanogaster*, auditory sensory systems do not appear to play a role in social organization (Table 5). Visual, gustatory, and olfactory manipulations cause flies to behave differently than wild-type flies in several ways (Schneider et al., 2012; Bentzur et al., 2020; Jiang et al., 2020; Rooke et al., 2020). However, these studies investigating the sensory mechanisms behind collective behavior are limited to unisex groups. It is possible that mixed groups of male and female flies may generate social structures that depend on a wider range of sensory systems since, for example, auditory cues are critical for courtship in flies (von Schilcher, 1976). Future studies should consider manipulating the composition of the social groups when inhibiting genes of interest to widen our knowledge of *Drosophila* social structures, like how Rooke et al. (2020) studied flies with *lush* inhibition at a variety of group sizes. It remains difficult to define how differences in precise network measures of mutants translate to differences in social organization, especially since various social network experiments utilize different methods of generating and analyzing networks. However, experiments that focused on social attraction and aggregation of flies used similar mutants and transgenic tools and found overlapping results to social network studies (Bentzur et al., 2020; Jiang et al., 2020; Sun et al., 2020). For example, Sun et al. (2020) reported that a combination of both vision and olfaction are crucial for the social attraction behavior of flies. This suggests that social networks capture some aspect of group-level social organization that is genetically and neurologically controlled. Recent work has demonstrated that the *for* gene plays a role in the social organization of adult flies since different polymorphisms are associated with differences in social network structure and manipulating the *for* gene influences this structure (Alwash et al., 2021). Further experimentation in social attraction and aggregation of flies at the neuronal and genetic level can assist in unraveling how abstract social network measures translate to a real-world group structure.

#### Social Transmission

To date, one group analyzed *Drosophila* social networks to directly study information flow, like many social network studies on ant colonies (Blonder and Dornhaus, 2011; Mersch et al., 2013; Stroeymeyt et al., 2018). Social communication within *Drosophila* groups can inform naïve flies about the presence of oviposition sites (Battesti et al., 2014) and the presence of predatory insects (Kacsoh et al., 2018). Pasquaretta et al. (2016a) applied SNA to examine how information spreads within a group of informed and naïve flies. This was done by video recording a 4-h training phase designed to inform focal flies of an oviposition site. Then social networks were generated within groups consisting of 8 informed flies and 4 uninformed flies (Pasquaretta et al., 2016a). Static, directed, and weighted social networks were generated every 15 min from 4 h of video footage. Afterward, every trained and untrained female fly was subjected to an oviposition site choice assay to determine if the mean and variance of social network measures predict whether uninformed flies follow or avoid the choices made by informed flies. Uninformed flies followed the correct choice when informed flies had less variable network distances from other individuals, as measured by weighted closeness centrality (defined in Table 1). Uninformed flies also followed when informed flies had a similar number of social contacts, as measured by eigenvector centrality (defined in Table 1) and when informed flies exchanged information to a similar extent, as measured by information centrality index (defined in Table 1). On the contrary, uninformed flies were less likely to follow the correct choice when they had a high betweenness centrality in the social network. Taken together, this suggests that when informed flies participate in most social interactions within the group, the uninformed flies are more likely to follow, and information is passed from the informed to the uninformed flies. In groups where uninformed flies were central to group cohesion (high betweenness centrality), the informed flies had less influence in transmitting the site preference. The authors also reported a remarkable finding where informed flies were more likely to avoid the media they were trained to prefer if they formed clusters, measured by a higher mean clustering coefficient. Properties of a social group are complex, and this highlights how individual foraging preferences can shift based on social associations within a group.

Diseases can also be transmitted via social interactions within a group. Utilizing the SNA approach in bumblebee colonies, for example, shed light on the relationship between interaction rate and parasitic transmission (Otterstatter and Thomson, 2007; Naug, 2008). So far, no studies to date have used SNA to explore how social interaction and network properties affect disease transmission in *Drosophila.* However, one study by Dawson et al. (2018) investigated how the social environment affects cancer progression in flies. In a homogenous group, cancerous flies were found to have higher interaction rate and duration than in heterogeneous groups consisting of cancerous and healthy flies. Additionally, Dawson et al. (2018) showed that tumor progression is slower when cancerous flies are kept in a homogenous group, and tumor progression is faster when cancerous flies are in isolation or within a group of healthy individuals. The use of the SNA approach can allow us to investigate the relationship between disease progression and social interactions even further by analyzing global network measures.

#### Evolution of Social Organization

Recently, a social network comparative study was conducted on 20 drosophilid species. Generating iterative, directed, and unweighted networks from groups of 12 male flies and groups of 12 female flies across all species, Jezovit et al. (2020) found no phylogenetic patterns for the species differences observed in assortativity, clustering coefficient, betweenness centrality, and global efficiency. This mirrors the results of a social network comparative analysis conducted on primates that also reported no evidence of phylogenetic signal in species-specific social networks (Pasquaretta et al., 2014). However, significant phylogenetic signal was found for the variation observed in social distance Jezovit et al. (2020). Social distance also correlated with the relative leg length of each species, suggesting morphological traits can influence behavioral evolution in flies. Next, the authors extracted averaged climate data from the geographic range of each drosophilid species and tested for correlations with each species’ averaged social network score. The authors found that variation in the climate data predicted species differences in the social network measures better than the differences found in the flies’ general behavioral characteristics such as average locomotor activity, average interaction duration, and average tendency to reciprocate interactions. Considering that each fly species descended from an inbred stock domesticated to the laboratory environment, it is surprising that factors of each species’ environment predicted differences in their social network measures. From these findings, we hypothesize that group-level organization is a behavioral trait that adapted to the abiotic selective pressures of each species’ habitat. For example, *Drosophila* species from tropical environments tend to have shorter cuticular hydrocarbons and rely more on visual sensory modalities than arid-adapted species (reviewed by Jezovit et al., 2017) and these ecological categories may also be relevant to species’ social structures measured by SNA. Finally, Jezovit et al. (2020) collected two independent datasets of social networks for 5 species, separated by 2 years at the time of collection. Consistent trends in the relative species’ differences were found for average assortativity, clustering coefficient, betweenness centrality, and global efficiency. This replication shows that species-specific social networks are robust and may represent phenotypes that emerge from physiological and behavioral mechanisms in individual flies.

Another recent comparative study by Wice and Saltz (2021) investigated the evolutionary relevance of social network measures across 20 different *D. melanogaster* strains. The authors generated static and directed social networks from mixed groups of flies using fixed criteria (see Table 2). These groups consisted of 10 males and 10 females, and each individual was genotypically distinct. The authors measured in-strength, out-strength, betweenness centrality, clustering coefficient, and eigenvector centrality for each fly within the group, and then compared the distribution of these measures for each genotype. This study stands out from other social network studies in that the authors were focused on measuring the characteristics of individual nodes and not the overall network structure. By comparing average network measures across numerous strains, the authors reported the broad sense heritability measure of clustering coefficient, betweenness centrality, and eigenvector centrality. Interestingly, betweenness centrality displayed the highest broad sense heritability score where genotypic differences account for 16.6% of the variation in this network measure (Wice and Saltz, 2021). This corroborates a prediction made by Schneider et al. (2012) that betweenness centrality may be a heritable trait based on robust differences observed between two *D. melanogaster* strains. To study environmental effects on social network measures, the authors reared the flies in various environments differing in calorie concentration and in the ratio of protein to carbohydrate content. The authors found no effect of environmental variation on betweenness centrality, similar to what Jezovit et al. (2020) found when comparing social networks across multiple *Drosophila* species. There is also evidence that various drosophilid species maintain consistent group structures across separate experiments, reinforcing the idea that social networks are emergent properties built from some genetic foundation shared by the individuals in the group (Jezovit et al., 2020). This view is strengthened by emerging evidence of specific genes accounting for differences in social networks within *Drosophila* (Bentzur et al., 2020; Rooke et al., 2020; Alwash et al., 2021). If social networks measure some heritable aspect of social behavior, then we can begin to consider that these properties are phenotypes that diversified through evolutionary selection mechanisms.

## Future Directions

Throughout this review, we have outlined experiments that all suggest *Drosophila* insects form organized and reproducible social networks when individuals aggregate. Despite *Drosophila* having long been considered solitary, a variety of organized collective behaviors have been uncovered in recent years. These collective behaviors provide a conceptual understanding of how social networks may function in fly groups. For example, flies in groups collectively escape from environmental threats (Ramdya et al., 2015) and enhance the survival of offspring through communal oviposition (Lihoreau et al., 2016). Oviposition site choice is influenced by social interaction with conspecifics. Battesti et al. (2012) demonstrated that when “teacher flies” are trained to deposit eggs on one of two food options, naïve “student” flies follow the same choice as the teachers after socially interacting. In addition, female flies arrest oviposition upon detection of predatory threats and can transmit this response to flies unaware of the threat (Kacsoh et al., 2015). Furthermore, flies in smaller group sizes exhibit a higher tendency to freeze their movement upon the detection of a predator (Ferreira and Moita, 2020), emphasizing the fitness benefits individuals gain from group formation. While it is unclear whether flies transmit information to one another, the above studies indicate that social interactions can lead to flies becoming informed of a stimulus, and ‘information transfer’ is a convenient term to describe this phenomenon. Applying SNA to these behavioral studies offers the opportunity to explore this concept of information transmission more precisely, and how other factors such as group size, density, and individual status contribute to the group-level output. So far one study applied SNA methods to study the oviposition site-choice phenomenon. The authors found that oviposition site choice influence from teacher flies are inhibited when student flies have stronger social ties in the group (Pasquaretta et al., 2016a). Interactions shared between flies appears to influence fitness-enhancing behaviors and this process can be visualized with networks.

Across animal social network studies, it is often reported that individuals maintain fixed positions in a social network over time (Brent et al., 2013; Krause et al., 2017; Blaszczyk, 2018; Stanley et al., 2018a; Canteloup et al., 2020). Other studies have reported a different view that individuals shift roles to maintain the stability of their social group and this flexibility maintains the group after individuals are lost due to predation and other stresses (Naug, 2008; Goldenberg et al., 2016; Firth et al., 2017; Formica et al., 2017). In flies there is evidence that the network position of individuals (degree) fluctuates, but the overall network structure of the group remains fixed over time (Schneider et al., 2012). A similar finding was reported in ants where an individual’s degree offered no predictive power over their degree later in the experiment (Blonder and Dornhaus, 2011). When studying animal groups in a controlled laboratory environment, there is evidence suggesting that individuals may not maintain fixed positions within social groups. This serves as an example how studying social networks in flies can enrich the broader animal social network literature. Areas of debate in these fields could be settled through social network experimentation in flies where vast resources are available to manipulate the organism genetically and physiologically, and large datasets can be acquired in controlled conditions.

The broader animal social network literature would also benefit from more studies manipulating the social environment of animals in controlled ways. In this review we outlined studies that examined social networks with manipulated group size and density (Rooke et al., 2020) and social networks from mixed groups of individuals with various social experiences (Pasquaretta et al., 2016a; Bentzur et al., 2020; Wice and Saltz, 2021). Future studies in *Drosophila* social networks should consider studying even more complex, mixed social environments. For instance, Jezovit et al. (2020) found that male-only social networks differ from female-only social networks in some species. Would mixing the sexes provide an intermediate social network phenotype, or could some interaction effect be observed? Wice and Saltz (2021) demonstrated females tend to occupy different social network positions than males when both sexes are mixed into the same social groups, but the authors did not attempt to study social organization of the group as a whole. Future experiments could analyze various layers of mixed-sex networks by generating separate networks from numerous criteria. Social space criteria can be refined to measure courtship and mating interactions or aggressive interactions. How would the properties of courtship and aggression networks compare to the properties of the general social networks? Experiments on courtship networks exist in the broader animal social network literature (Ryder et al., 2008; Oh and Badyaev, 2010; Formica et al., 2012; Fisher et al., 2016) and it would be worthwhile to determine if the *Drosophila* courtship networks overlap with these other studies.

Finally, *Drosophila* has a long history of serving as a model organism for the genetic basis of social behavior. Applying social network methods to screen well-studied mutants may aid in uncovering genetic mechanisms of sociality. For instance, a recent study found a potential role the *foraging* gene plays in the collective behavior of flies that can be measured using social networks (Alwash et al., 2021). Heritable factors in social network measures has also been reported in humans, rhesus macaques, and flies (Fowler et al., 2009; Brent et al., 2013; Wice and Saltz, 2021), reinforcing the idea that robust social network measures represent phenotypes of collective group structures. Although there is evidence that social network phenotypes do not map well onto phylogenetic trees (Jezovit et al., 2020), it does not rule out that these social behaviors have no underlying and conserved biological mechanisms. The circadian clock is one example of a conserved biological system that is pervasive across various organisms, yet circadian rhythms as a behavior vary across organisms from different habitats (Dunlap et al., 2004; Sehgal, 2015). Further experimental efforts using *Drosophila* and the vast genetic tools available within this system could uncover genetic and neurological mechanisms governing collective behavior. These findings may one day contribute toward identifying ancient mechanisms of sociality similar to how other pervasive mechanisms, like the circadian clock, have been uncovered in *Drosophila.*

## Author Contributions

JJ and NA wrote the first draft and planned the manuscript with JL. JJ and NA designed the figures. All authors edited the manuscript.

## Conflict of Interest

The authors declare that the research was conducted in the absence of any commercial or financial relationships that could be construed as a potential conflict of interest.

## Publisher’s Note

All claims expressed in this article are solely those of the authors and do not necessarily represent those of their affiliated organizations, or those of the publisher, the editors and the reviewers. Any product that may be evaluated in this article, or claim that may be made by its manufacturer, is not guaranteed or endorsed by the publisher.
